# Influenza vaccination uptake and associated factors among healthcare workers in Chengdu’s maternal and child health institutions in the post-pandemic era

**DOI:** 10.3389/fpubh.2026.1766101

**Published:** 2026-04-13

**Authors:** Mei Cha, Hongmei Tang, Hairong Wang, Qiaoqiao Li, Erdan Luo, Qin Tang, Wenjie Qing, Wei He, Dingqian Gu, Dingyu Yan, Jing Shu, Yuerong Ding, Yao Sun, Xiu Chen, Yan Xiang, Yongmei Zhao, Yunxian Jiang, Jingqiu Xie, Bei Wang, Jinghua Ye, Wenxu Yang, Yi Yang, Qin Zhang, Yonghong Lin

**Affiliations:** 1 Chengdu Women's and Children's Central Hospital, School of Medicine, University of Electronic Science and Technology of China, Chengdu, Sichuan, China; 2 Chengdu Fifth People's Hospital (The Second Clinical Medical College Affiliated Fifth People's Hospital of Chengdu University of Traditional Chinese Medicine), Chengdu, Sichuan, China; 3 Jianyang Center for Disease Control and Prevention, Chengdu, Sichuan, China; 4 Pengzhou Maternity and Child Healthcare Hospital, Pengzhou Women’s and Children’s Hospital, Chengdu, Sichuan, China; 5 Shuangliu Maternal and Child Health Hospital, Chengdu, Sichuan, China; 6 Longquanyi District of Chengdu Maternity & Child Health Care Hospital, Chengdu, Sichuan, China; 7 Chengdu Xindu Maternal and Child Health Care Hospital, Chengdu, Sichuan, China; 8 Xinjin Branch of Chengdu Women's and Children's Central Hospital, Chengdu, Sichuan, China; 9 Jingtang MCH Hospital, Chengdu, Sichuan, China; 10 Dayi County Women and Children's Hospital, Chengdu, Sichuan, China; 11 Jiangdu Obstetric and Gynecology Hospital, Chengdu, Sichuan, China; 12 Chengdu Qingyang Women's and Children's Health Care and Family Planning Service Center (Chengdu Qingyang Women and Children's Health Hospital), Chengdu, Sichuan, China

**Keywords:** healthcare workers, influenza vaccination, maternal and child health institutions, post-pandemic period, vaccine hesitancy

## Abstract

**Purpose:**

This study assessed influenza vaccination uptake, knowledge, beliefs, and willingness among healthcare workers (HCWs) in maternal and child health (MCH) institutions in Chengdu in the post-COVID-19 era and identified factors associated with vaccination behavior.

**Methods:**

A cross-sectional online survey was conducted in 10 Chengdu MCH institutions from May 12 to June 30, 2025, using a validated questionnaire. Analyses included descriptive statistics, univariate tests (chi-square; independent-samples *t*-tests/one-way ANOVA), and hierarchical multivariable logistic regression.

**Results:**

Among 1,880 HCWs, 35.21% (662) reported receiving influenza vaccination since June 2024, and 10.43% reported clinician-diagnosed influenza. Before the 2025 influenza season, 57.61% reported willingness to be vaccinated. Objective knowledge was suboptimal: 45.05% correctly identified the time to develop protective antibodies (2–4 weeks), and 43.72% correctly identified the duration of protection (at least 6 months). Belief scores increased with higher education and professional title (*p* < 0.05) and were lowest among outsourced staff. Hierarchical regression (Nagelkerke *R*^2^ = 0.377) identified independent correlates of vaccination uptake, including older age [odds ratio (OR) = 1.029 per year], master’s degree or above (OR = 2.067), working in secondary-level MCH hospitals (OR = 5.377) or outpatient/preventive-care departments (OR = 1.682), being a nurse (OR = 1.804) or administrative staff member (OR = 3.262) versus physician, higher belief score (OR = 1.062 per point), and, most strongly, willingness to vaccinate (uncertain vs. unwilling: OR = 3.431; willing vs. unwilling: OR = 23.296).

**Conclusion:**

Influenza vaccination coverage among HCWs in Chengdu MCH institutions remained suboptimal in the post-pandemic period, with a clear intention-behavior gap. Uptake was associated with more favorable beliefs, higher willingness, older age, higher education, and institutional/departmental and occupational factors. Targeted strategies to strengthen confidence, improve access and convenience, and tailor interventions to specific departments and job categories are needed to increase coverage.

## 1 Introduction

Seasonal influenza contributes to substantial morbidity and healthcare utilization each year and imposes a considerable economic burden. Healthcare workers are at elevated risk of influenza infection because of frequent occupational exposure (1–3). Maternal and child health (MCH) institutions serve a high proportion of pregnant women, newborns, and young children; once influenza transmission occurs, it may spread rapidly within these susceptible populations. National and international guidelines consistently identify healthcare workers as a priority group for influenza vaccination (4, 5). Vaccination can reduce influenza infection and influenza-like illness, limit nosocomial transmission, and provide indirect protection to maternal and infant populations (6, 7).

In the post-COVID-19 period, infection prevention practices and risk perceptions in healthcare settings have shifted. At the same time, vaccine fatigue, opportunity-cost considerations, and risk reassessment may reduce motivation for seasonal vaccination (8–10). In western China, systematic quantitative evidence on influenza vaccination status, knowledge, beliefs, and behavioral intentions among healthcare workers in MCH institutions remains limited. Prior studies have largely focused on general hospitals or specific specialties, making it difficult to characterize the organizational context of MCH institutions where outpatient and preventive-care departments constitute a large proportion and pediatrics and obstetrics/gynecology coexist. Moreover, pregnant women are a key population recommended for influenza vaccination, and healthcare workers’ perceptions of vaccination during pregnancy may influence clinical communication and decision-making; however, data in this domain are insufficient.

Therefore, we conducted a cross-sectional survey in MCH institutions in Chengdu to describe healthcare workers’ influenza infection and vaccination status, assess their knowledge, beliefs and attitudes, and willingness to vaccinate, and identify factors associated with vaccination behavior. The findings aim to provide quantitative evidence for regional seasonal influenza prevention and control.

## 2 Materials and methods

### 2.1 Study design and setting

Healthcare workers from 10 maternal and child health (MCH) institutions in Chengdu were invited to participate. An anonymous online survey was administered using the Wenjuanxing platform. From May 12 to June 30, 2025, the total number of eligible on-duty healthcare workers across the participating institutions was 4,395. A total of 2,043 questionnaires were submitted (overall response rate: 46.48%). After applying predefined quality-control criteria, 1,880 questionnaires were retained for analysis (valid response rate: 92.02%).

Inclusion criteria were: (1) full-time or contract employment at one of the participating MCH institutions during the survey period; (2) age 18 years or older; (3) job categories including physicians, nurses, medical-technical personnel, administrative staff, logistics personnel, outsourced staff, and other related roles; and (4) ability to read Chinese and complete the questionnaire via a smartphone.

Exclusion criteria were: (1) interns, trainees, or temporary personnel not formally employed by the institution; (2) individuals not employed at the institution throughout the survey period; and (3) questionnaires not meeting quality-control standards, including duplicate submissions identified by the platform backend, missing data for the primary outcome or substantial missingness in key variables, illogical or inconsistent responses, and completion times deemed implausibly short (suggesting inattentive responding).

### 2.2 Questionnaire development and validation

A questionnaire was developed after a literature review and iterative item refinement. Four experts (three clinical healthcare professionals and one hospital administrator) evaluated content validity using a five-point Likert scale across five dimensions: relevance, comprehensiveness, representativeness, appropriateness of response options, and ethical adherence. The item-level content validity index (I-CVI) for each item was 0.80 or higher, and the scale-level content validity index (S-CVI) was 0.95.

A pilot survey was conducted among 100 healthcare workers prior to the formal survey. Construct validity was supported by the Kaiser-Meyer-Olkin (KMO) test and Bartlett’s test of sphericity (KMO = 0.805; Bartlett’s chi-square = 263.077, *p* < 0.001). The questionnaire demonstrated acceptable internal consistency (Cronbach’s alpha = 0.807 for standardized items).

The formal survey link was distributed via institutional WeChat working groups of the participating institutions. Participation was voluntary. The Medical Ethics Committee of Chengdu Women’s and Children’s Central Hospital approved the study [Approval No. 2025(21)2].

The questionnaire comprised 28 items covering five domains: influenza infection status, influenza vaccination status, knowledge of influenza vaccination, willingness to vaccinate, and attitudinal beliefs. Beliefs regarding influenza and influenza vaccination were assessed in two dimensions (perceived disease risk and perceived value of vaccination) using five positively worded items on a five-point Likert scale (fully agree = 5 to fully disagree = 1). Total scores ranged from 5 to 25, with higher scores indicating more favorable beliefs about influenza vaccination.

### 2.3 Quality control

A survey team consisting of two public health experts and two key research staff members oversaw implementation and data quality. During data cleaning, questionnaires were excluded according to predefined quality-control rules, including failure to meet inclusion criteria, obvious logical inconsistencies, implausibly short completion time, uniform response patterns across multiple items, and excessive missing data in key variables.

### 2.4 Statistical analysis

IBM SPSS Statistics 29 was used to establish the database and perform analyses. Questionnaire reliability and validity were evaluated using Cronbach’s alpha, the KMO test, and Bartlett’s test of sphericity. Continuous variables are presented as mean ± standard deviation (SD). Categorical variables are presented as frequency and percentage (n, %).

Chi-square tests were used to compare influenza infection rates, vaccination rates, knowledge awareness, and willingness to vaccinate across demographic groups. For categorical variables with statistically significant overall differences, *post hoc* pairwise comparisons were performed using Bonferroni-adjusted *p* values. Belief scores were compared across demographic groups using independent-samples *t* tests (two groups) or one-way ANOVA (three or more groups). Depending on variance homogeneity, post hoc comparisons were conducted using the Student–Newman–Keuls (SNK) test or Dunnett’s t test.

Before hierarchical logistic regression, multicollinearity among candidate predictors was assessed. Pearson correlation coefficients were calculated for continuous variables, and variance inflation factors (VIFs) were computed using a linear regression model that included all candidate covariates. Based on these diagnostics and theoretical considerations, non-significant variables that were highly correlated with other predictors were excluded from the final hierarchical model. A two-sided *p*-value < 0.05 was considered statistically significant.

## 3 Results

### 3.1 Basic characteristics of the study population and current status of vaccination

#### 3.1.1 Participant characteristics

The mean age of respondents was 37.94 years. Most participants were female (88.30%), and the majority held a bachelor’s degree (68.62%). Nurses (42.50%) and physicians (27.77%) were the largest occupational groups. Most participants worked in a tertiary MCH specialty hospital (93.14%). The most common departments were outpatient and preventive-care (20.00%), pediatrics (19.52%), obstetrics/gynecology (15.85%), administrative/logistics (14.47%), and medical-technical departments (12.77%). The mean duration of employment was 13.14 years, and the most common professional titles were intermediate (41.59%) and junior (33.78%) (Table 1).

**Table 1:** Demographic characteristics of participants (*N* = 1,880).

Characteristic	*n* (%)
Sex	
Female	1,660 (88.30)
Male	220 (11.70)
Age (years)	
≤30	455 (24.20)
31–40	796 (42.34)
41–50	401 (21.33)
≥51	228 (12.13)
Education level	
Junior college or below	360 (19.15)
Bachelor’s degree	1,290 (68.62)
Master’s degree or above	230 (12.23)
Job category	
Physician	522 (27.77)
Nurse	799 (42.50)
Medical-technical staff	202 (10.74)
Administrative staff	210 (11.17)
Outsourced staff (e.g., cleaners)	124 (6.60)
Other	23 (1.22)
Hospital level/type	
Tertiary MCH specialty hospital	1,751 (93.14)
Secondary MCH specialty hospital	129 (6.86)
Department	
Obstetrics & gynecology	298 (15.85)
Pediatrics	367 (19.52)
Outpatient & preventive-care departments	376 (20.00)
Emergency & perioperative departments	185 (9.84)
Medical-technical departments	240 (12.77)
Administrative & logistics departments	272 (14.47)
Other departments	142 (7.55)
Years of employment	
≤10	898 (47.76)
11–20	616 (32.77)
21–30	257 (13.67)
≥31	109 (5.80)
Professional title	
Senior	281 (14.95)
Intermediate	782 (41.59)
Junior	635 (33.78)
None	182 (9.68)

MCH, maternal and child health.

#### 3.1.2 Influenza infection in the past year

From June 2024 to the survey date, 196 participants (10.43%) reported clinician-diagnosed influenza. Among them, 47 were pathogen-positive, 43 were pathogen-negative, and 106 were clinically diagnosed without pathogen testing. In addition, 477 participants (25.37%) reported influenza-like symptoms but did not seek medical care or receive a clinical diagnosis. Overall, 64.20% reported no influenza-like symptoms. Participants who reported clinician-diagnosed influenza and those who reported influenza-like symptoms without clinical diagnosis were combined as having influenza-like illness (ILI) symptoms (*n* = 673). The proportion with ILI symptoms differed across departments (*p* < 0.05), with higher percentages observed in outpatient/preventive-care, emergency/perioperative, and administrative/logistics departments than in other departments (Table 2). Among the 673 participants with ILI symptoms, 363 (53.94%) reported symptom onset before vaccination and 310 (46.06%) reported symptom onset after vaccination.

**Table 2:** Distribution of participants with influenza-like illness symptoms by characteristics (*N* = 1,880).

Characteristic	Total	ILI symptoms *n* (%)	*χ* ^2^	*p*
Sex				
Female	1,660	594 (35.78)		
Male	220	79 (35.91)	0.001	0.971
Age (years)				
≤30	455	165 (36.26)		
31–40	796	295 (37.06)		
41–50	401	140 (34.91)		
≥51	228	73 (32.02)	2.149	0.542
Education level				
Junior college or below	360	122 (33.89)		
Bachelor’s degree	1,290	466 (36.12)		
Master’s degree or above	230	85 (36.96)	0.765	0.682
Job category				
Physician	522	194 (37.16)		
Nurse	799	273 (34.17)		
Medical-technical staff	202	74 (36.63)		
Administrative staff	210	89 (42.38)		
Outsourced staff (e.g., cleaners)	124	33 (26.61)		
Other	23	10 (43.48)	10.511	0.062
Hospital level/type				
Tertiary MCH specialty hospital	1,751	629 (35.92)		
Secondary MCH specialty hospital	129	44 (34.11)	0.172	0.678
Department				
Obstetrics and gynecology	298	105 (35.23)		
Pediatrics	367	114 (31.06)		
Outpatient and preventive-care departments	376	149 (39.63) ^a^		
Emergency and perioperative departments	185	75 (40.54) ^b^		
Medical-technical departments	240	84 (35.00)		
Administrative and logistics departments	272	110 (40.44) ^c^		
Other departments	142	36 (25.35)	17.191	**0.009**
Years of employment				
≤10	898	344 (38.31)		
11–20	616	198 (32.14)		
21–30	257	93 (36.19)		
≥31	109	38 (34.86)	6.100	0.107
Professional title				
Senior	281	111 (39.50)		
Intermediate	782	279 (35.68)		
Junior	635	216 (34.02)		
None	182	67 (36.81)	2.641	0.450

ILI, influenza-like illness. MCH, maternal and child health.

^a^ The ILI symptoms proportion of outpatient and preventive-care departments (39.63%) was higher than the other departments (25.35%) (*χ*^2^ = 9.149, adjusted *p* = 0.042).

^b^ The ILI symptoms proportion of emergency and perioperative departments (40.54%) was higher than the other departments (25.35%) (with *χ*^2^ = 8.265, adjusted *p* = 0.028).

^c^ The ILI symptoms proportion of administrative and logistics departments (40.44%) was higher than the other departments (25.35%) (*χ*^2^ = 9.305, adjusted *p* = 0.014).

Bold values indicate statistical significance (*p* < 0.05).

#### 3.1.3 Influenza vaccination and adverse events in the past year

From June 2024 to the survey date, 662 participants reported receiving influenza vaccination, yielding an uptake of 35.21%. Uptake in participants aged 30 years or younger was lower than that in those aged 31–40 and 41–50 years. Participants with junior college education or below had lower uptake than those with bachelor’s or master’s degrees. Uptake differed by job category; administrative staff had higher uptake than physicians, nurses, and outsourced staff. Uptake in tertiary MCH specialty hospitals was lower than that in secondary MCH specialty hospitals. Uptake also varied by department: obstetrics/gynecology and emergency/perioperative departments had lower uptake than outpatient/preventive-care and administrative/logistics departments, and pediatrics had lower uptake than outpatient/preventive-care departments. Participants with 10 years of employment or less had lower uptake than those with 11–20 or 21–30 years, and those with junior professional titles had lower uptake than those with senior titles (Table 3).

**Table 3:** Influenza vaccination uptake across demographic subgroups (*N* = 1,880).

Characteristic	Total	Vaccinated *n* (%) (*N* = 662)	*χ* ^2^	*p*
Sex				
Female	1,660	579 (34.88)		
Male	220	83 (37.73)	0.691	0.406
Age (years)				
≤30	455	125 (27.47) ^a^		
31–40	796	299 (37.56)		
41–50	401	154 (38.40)		
≥51	228	84 (36.84)	15.931	**0.001**
Education level				
Junior college or below	360	105 (29.17) ^b^		
Bachelor’s degree	1,290	467 (36.20)		
Master’s degree or above	230	90 (39.13)	7.869	**0.020**
Job category				
Physician	522	182 (34.87)		
Nurse	799	264 (33.04)		
Medical-technical staff	202	74 (36.63)		
Administrative staff	210	103 (49.05) ^c^		
Outsourced staff (e.g., cleaners)	124	32 (25.81)		
Other	23	7 (30.43)	24.516	**<0.001**
Hospital level/type				
Tertiary MCH specialty hospital	1,751	571 (32.61) ^d^		
Secondary MCH specialty hospital	129	91 (70.54)	75.780	**<0.001**
Department				
Obstetrics and gynecology	298	81 (27.18) ^e^		
Pediatrics	367	122 (33.24) ^f^		
Outpatient and preventive-care departments	376	166 (44.15) ^g^		
Emergency and perioperative departments	185	53 (28.65) ^h^		
Medical-technical departments	240	88 (36.67) ^i^		
Administrative and logistics departments	272	121 (44.49) ^j^		
Other departments	142	31 (21.83)	47.326	**<0.001**
Years of employment				
≤10	898	271 (30.18) ^k^		
11–20	616	244 (39.61)		
21–30	257	103 (40.08)		
≥31	109	44 (40.37)	19.135	**<0.001**
Professional title				
Senior	281	121 (43.06) ^l^		
Intermediate	782	301 (38.49) ^m^		
Junior	635	187 (29.45)		
None	182	53 (29.12)	23.478	**<0.001**

MCH, maternal and child health.

^a^ The influenza vaccination uptake proportion of ≤30 years old (27.47%) was lower than 31–40 years old (37.56%) (*χ*^2^ = 13.156, adjusted *p* < 0.05), 41–50 years old (38.40%) (*χ*^2^ = 11.593, adjusted *p* < 0.05) and ≥51 years old (36.84%) (*χ*^2^ = 6.279, adjusted *p* = 0.048).

^b^ The influenza vaccination uptake proportion of junior college or below (29.17%) was lower than bachelor’s degree (36.20%) (*χ*^2^ = 6.150, adjusted *p* = 0.039) and master’s degree or above (39.13%) (*χ*^2^ = 6.296, adjusted *p* = 0.036).

^c^ The influenza vaccination uptake proportion of administrative staffs (49.05%) was higher than physicians (34.87%) (*χ*^2^ = 12.668, adjusted *p* < 0.05), nurse (33.04%) (*χ*^2^ = 18.409, adjusted *p* < 0.05), outsourced staff (25.81%) (*χ*^2^ = 17.487, adjusted *p* < 0.05).

^d^ The influenza vaccination uptake proportion of tertiary MCH specialty hospital (32.61%) was lower than secondary MCH specialty hospital (70.54%) (*χ*^2^ = 75.780, *p* < 0.001).

^e^ The influenza vaccination uptake proportion of obstetrics and gynecology (27.18%) was lower than outpatient and preventive-care departments (44.15%) (*χ*^2^ = 20.615, adjusted *p* < 0.05) and administrative and logistics departments (44.49%) (*χ*^2^ = 18.611, adjusted *p* < 0.05).

^f^ The influenza vaccination uptake proportion of pediatrics (33.24%) was lower than outpatient and preventive-care departments (44.15%) (*χ*^2^ = 9.307, adjusted *p* = 0.014) and administrative and logistics departments (44.49%) (*χ*^2^ = 8.379, adjusted *p* = 0.028).

^g^ The influenza vaccination uptake proportion of outpatient and preventive-care departments (44.15%) was higher than emergency and perioperative departments (28.65%) (*χ*^2^ = 12.518, adjusted *p* < 0.05) and the other departments (21.83%) (*χ*^2^ = 21.784, adjusted *p* < 0.05).

^h^ The influenza vaccination uptake proportion of emergency and perioperative departments (28.65%) was lower than administrative and logistics departments (44.49%) (*χ*^2^ = 11.712, adjusted *p* < 0.05).

^i^ The influenza vaccination uptake proportion of medical-technical departments (36.67%) was higher than the other departments (21.83%) (*χ*^2^ = 9.155, adjusted *p* = 0.014).

^j^ The influenza vaccination uptake proportion of administrative and logistics departments (44.49%) was higher than the other departments (21.83%) (*χ*^2^ = 20.135, adjusted *p* < 0.05).

^k^ The influenza vaccination uptake proportion of ≤10 years employment (30.18%) was lower than 11–20 years employment (39.61%) (*χ*^2^ = 14.482, adjusted *p* < 0.05) and 21–30 years employment (40.08%) (*χ*^2^ = 8.944, adjusted *p* = 0.012).

^l^ The influenza vaccination uptake proportion of senior title (43.06%) was higher than junior title (29.45%) (*χ*^2^ = 16.171, adjusted *p* < 0.05) and no title (29.12%) (*χ*^2^ = 9.150, adjusted *p* = 0.008).

^m^ The influenza vaccination uptake proportion of intermediate title (38.49%) was higher than junior title (29.45%) (*χ*^2^ = 12.690, adjusted *p* < 0.05).

Bold values indicate statistical significance (*p* < 0.05).

Among vaccinated participants, 91 (13.75%) reported post-vaccination discomfort. A total of 82 symptom reports were recorded because some participants reported multiple symptoms. Systemic reactions and injection-site reactions were most commonly reported (Table 4).

**Table 4:** Reported adverse events following influenza vaccination (*N* = 82).

Adverse event category	Reports n (%)	Typical descriptions (frequency)
Systemic reactions	39 (47.56)	Fatigue (1), myalgia (2), fever (3)
Injection-site reactions	35 (42.68)	Redness/swelling (3), local swelling/pain (4), injection-site pain (4), arm pain (5)
Other reactions	8 (9.76)	Cold-like symptoms (5), sore throat (4), allergy (5)

**Figure 1:**
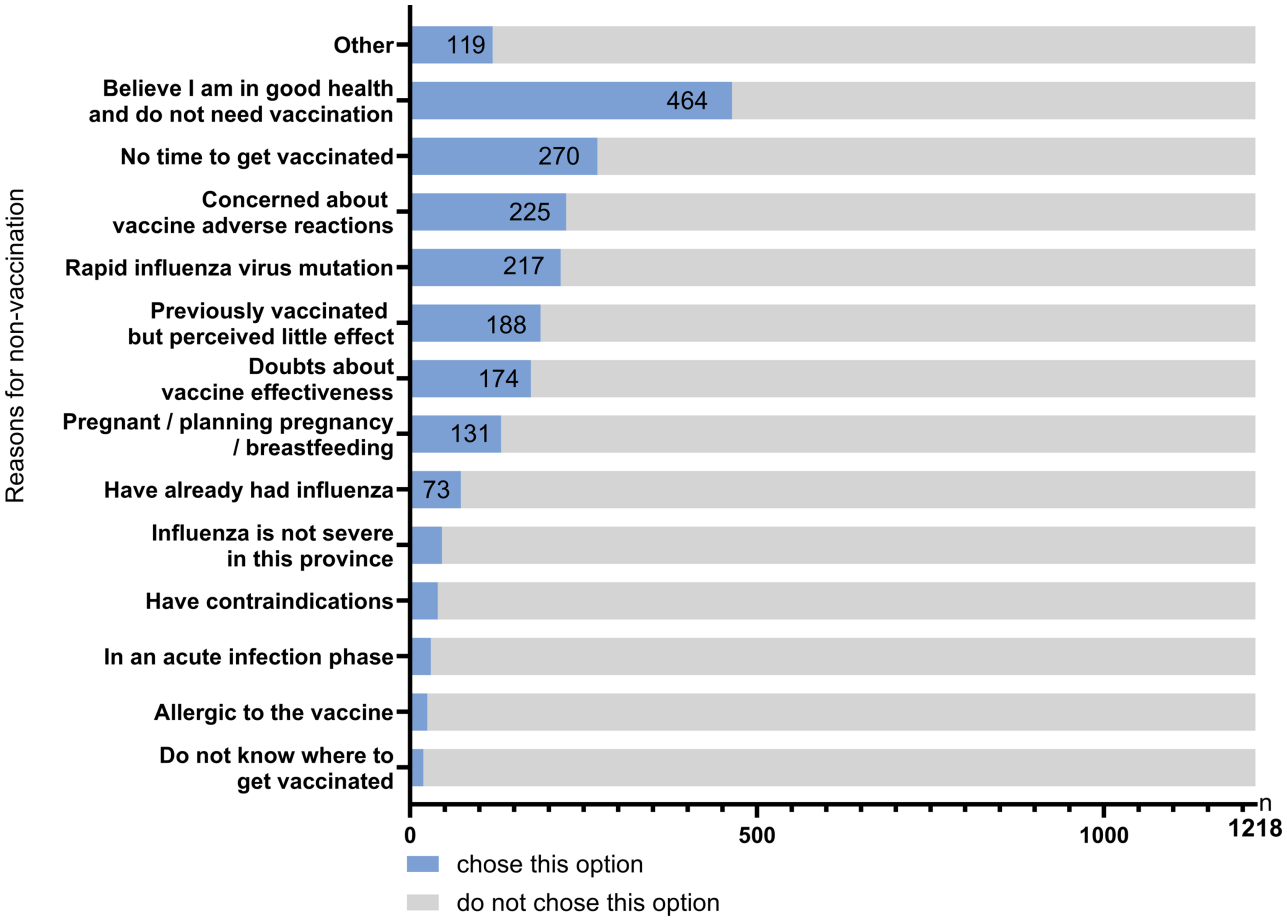
Reasons for not receiving influenza vaccination in 2024.

Among the 1,218 unvaccinated participants, respondents could endorse multiple reasons for not receiving vaccination. The most frequently reported reasons were perceiving themselves as healthy with no need for vaccination, lack of time, concerns about adverse reactions, belief that rapid viral mutation reduces vaccine effectiveness, and perceived lack of benefit from previous vaccination (Figure 1).

#### 3.1.4 Awareness of influenza vaccine-related knowledge

Influenza vaccine knowledge was assessed using both objective knowledge items and subjective self-rated awareness (Table 5). For the objective item on time to develop protective antibodies after vaccination, 847 participants (45.05%) correctly identified 2–4 weeks. For the duration of protection, 130 participants (6.91%) correctly selected 6–8 months; an additional 36.81% selected “6 months or at least 6 months.” When responses indicating protection for at least 6 months were combined, 822 participants (43.72%) were classified as aware of protection duration.

**Table 5:** Awareness of influenza vaccine-related knowledge (*N* = 1,880).

Influenza vaccine knowledge item	*n* (%)
Time to develop protective antibodies after vaccination	
Within 1 week	175 (9.31)
2–4 weeks	847 (45.05)
1 month	310 (16.49)
2 months	80 (4.26)
Uncertain	468 (24.89)
Duration of protection after vaccination	
6–8 months	130 (6.91)
6 months or ≥6 months	692 (36.81)
Other duration	755 (40.16)
Do not know	303 (16.12)
Awareness of different influenza vaccine types	
Yes, know differences between trivalent and quadrivalent vaccines	648 (34.47)
No, do not know vaccine types	411 (21.86)
Partially aware	821 (43.67)
Awareness of annual vaccine update	
Yes, aware vaccines updated annually based on viral drift	528 (28.08)
No, not aware of annual update	616 (32.77)
Partially aware	736 (39.15)
Awareness of China CDC 2024 Technical Guidelines	
Completely unaware	246 (13.09)
Slightly aware	512 (27.23)
Uncertain	285 (15.16)
Aware of some content	729 (38.78)
Fully aware	108 (5.74)

**Figure 2:**
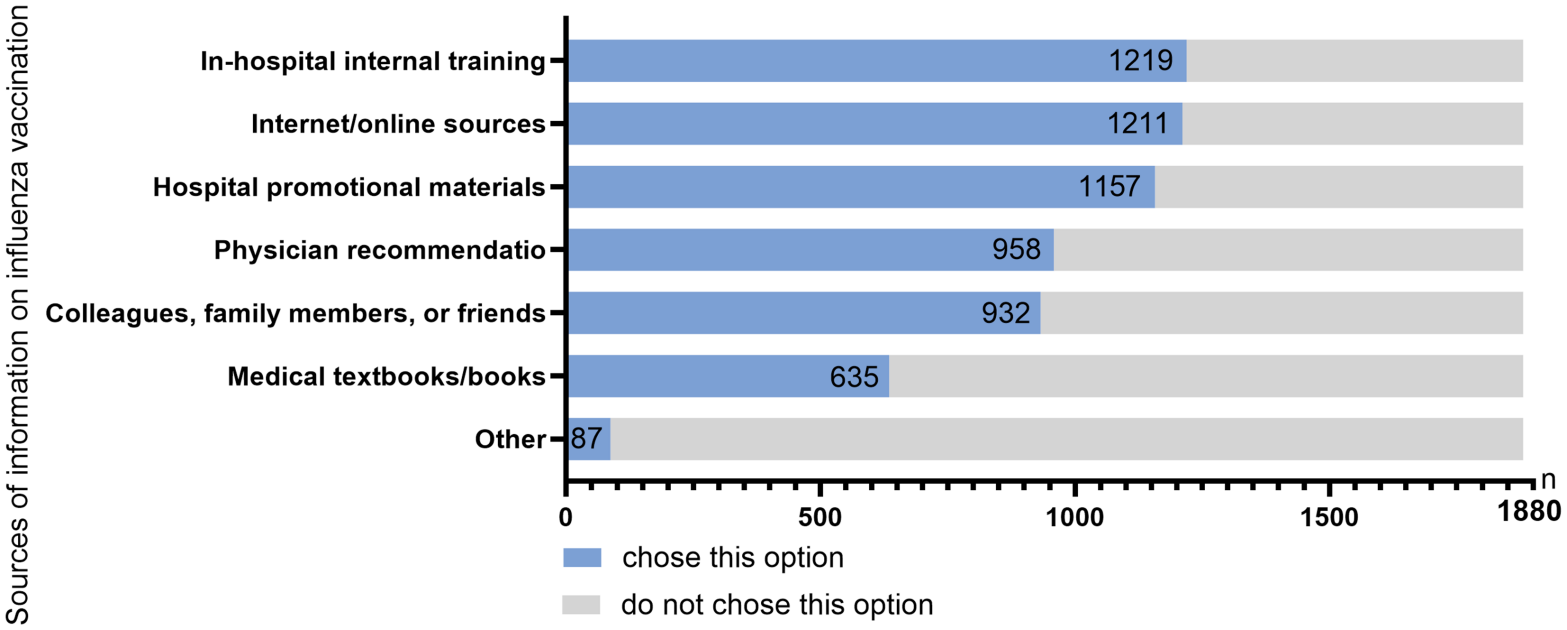
Sources of information on influenza vaccination.

For subjective awareness, 34.47% reported knowing the differences between trivalent and quadrivalent vaccines, and 43.67% reported partial awareness. Regarding annual vaccine updates, 28.08% reported full awareness and 39.15% partial awareness. Regarding the China CDC 2024 Technical Guidelines for Influenza Vaccination, only 5.74% reported full awareness and 38.78% reported partial awareness. Respondents reported multiple sources of vaccine-related information, including internal hospital training, online resources, hospital promotional materials, physician recommendations, colleagues, family and friends, and medical literature (Figure 2).

#### 3.1.5 Differences in objective knowledge mastery among subgroups

Because subjective self-assessments may not reflect actual proficiency, objective knowledge items were used to evaluate knowledge mastery. For time to develop protective antibodies, awareness was lower among females than males, lower among participants aged 51 years or older than those aged 41–50 years, and increased with higher educational attainment. Outsourced personnel (e.g., cleaners) had lower awareness than physicians, nurses, medical-technical staff, and administrative staff. Nurses and administrative staff had lower awareness than physicians. Differences were also observed across departments; in particular, awareness in obstetrics/gynecology was lower than that in outpatient/preventive-care and emergency/perioperative departments. Participants with 21–30 years of employment had higher awareness than those with 10 years or less or 11–20 years. Participants without a professional title had lower awareness than those with senior or intermediate titles.

For awareness of protection duration, participants aged 51 years or older had lower awareness than younger age groups; participants with junior college education or below had lower awareness than those with bachelor’s or master’s degrees. Outsourced staff had lower awareness than physicians, nurses, medical-technical staff, and administrative staff, and participants without professional titles had lower awareness than those with intermediate titles (Table 6).

**Table 6:** Objective knowledge of antibody development time and duration of protection by characteristics (*N* = 1,880).

Characteristic	Total	Aware of antibody development time (*N* = 847)			Aware of protection duration (*N* = 822)		
		*n* (%)	*χ* ^2^	*p*	*n* (%)	*χ* ^2^	*p*
Sex							
Female	1,660	734 (44.22) ^a^	4.008	**0.045**	725 (43.67)	0.014	0.907
Male	220	113 (51.36)			97 (44.09)		
Age (years)							
≤30	455	204 (44.84)			209 (45.93)		
31–40	796	360 (45.23)			372 (46.73)		
41–50	401	198 (49.38) ^b^			171 (42.64)		
≥51	228	85 (37.28)			70 (30.70) ^l^		
Education level							
Junior college or below	360	129 (35.83) ^c^	22.262	**<0.001**	129 (35.83) ^m^	14.333	**<0.001**
Bachelor’s degree	1,290	591 (45.81) ^d^			576 (44.65)		
Master’s degree or above	230	127 (55.22)			117 (50.87)		
Job category							
Physician	522	298 (57.09) ^e^	65.901	**<0.001**	254 (48.66)	35.245	**<0.001**
Nurse	799	327 (40.93) ^f^			354 (44.31)		
Medical-technical staff	202	99 (49.01) ^g^			94 (46.53)		
Administrative staff	210	87 (41.43) ^h^			84 (40.00)		
Outsourced staff (e.g., cleaners)	124	27 (21.77)			25 (20.16) ^n^		
Other	23	9 (39.13)			11 (47.83)		
Hospital level/type							
Tertiary MCH specialty hospital	1,751	782 (44.66)			764 (43.63)		
Secondary MCH specialty hospital	129	65 (50.39)			58 (44.96)		
Department							
Obstetrics and gynecology	298	113 (37.92) ^i^	16.617	**0.011**	117 (39.26)	10.491	0.105
Pediatrics	367	167 (45.50)			166 (45.23)		
Outpatient and preventive-care departments	376	184 (48.94)			168 (44.68)		
Emergency and perioperative departments	185	96 (51.89)			95 (51.35)		
Medical-technical departments	240	118 (49.17)			111 (46.25)		
Administrative and logistics departments	272	112 (41.18)			107 (39.34)		
Other departments	142	57 (40.14)			58 (40.85)		
Years of employment							
≤10	898	394 (43.88)	9.865	**0.020**	400 (44.54)	0.619	0.892
11–20	616	267 (43.34)			263 (42.69)		
21–30	257	139 (54.09) ^j^			113 (43.97)		
≥31	109	47 (43.12)			46 (42.20)		
Professional title							
Senior	281	146 (51.96)	14.335	**0.002**	118 (41.99)	11.248	**0.010**
Intermediate	782	361 (46.16)			371 (47.44) ^o^		
Junior	635	277 (43.62)			270 (42.52)		
None	182	63 (34.62) ^k^			63 (34.62)		

MCH, maternal and child health.

^a^ Awareness of antibody development proportion of female (44.22%) was lower than male (51.36%) (*χ*^2^ = 4.008, *p* = 0.045).

^b^ Awareness of antibody development proportion of 41–50 years old (49.38%) was higher than≥51 years old (37.28%) (*χ*^2^ = 8.593, adjusted *p* = 0.012).

^c^ Awareness of antibody development proportion of junior college or below (35.83%) was lower than bachelor’s degree (45.81%) (*χ*^2^ = 11.399, adjusted *p* < 0.05) and master’s degree or above (55.22%) (*χ*^2^ = 21.468, adjusted *p* < 0.05).

^d^ Awareness of antibody development proportion of bachelor’s degree (45.81%) was lower than master’s degree or above (55.22%) (*χ*^2^ = 6.925, adjusted *p* = 0.024).

^e^ Awareness of antibody development proportion of physician (57.09%) was higher than nurse (40.93%) (*χ*^2^ = 33.084, adjusted *p* < 0.05), administrative staff (41.43%) (*χ*^2^ = 14.729, adjusted *p* < 0.05) and outsourced staff (21.77%) (*χ*^2^ = 49.984, adjusted *p* < 0.05).

^f^ Awareness of antibody development proportion of nurse (40.93%) was higher than outsourced staff (21.77%) (*χ*^2^ = 16.653, adjusted *p* < 0.05).

^g^ Awareness of antibody development proportion of medical-technical staff (49.01%) was higher than outsourced staff (21.77%) (*χ*^2^ = 16.653, adjusted *p* < 0.05).

^h^ Awareness of antibody development proportion of outsourced staff (21.77%) was lower than administrative staff (41.43%) (*χ*^2^ = 13.396, adjusted *p* < 0.05).

^i^ Awareness of antibody development proportion of obstetrics & gynecology (37.92%) was lower than outpatient and preventive-care departments (48.94%) (*χ*^2^ = 8.186, adjusted *p* = 0.028), emergency and perioperative departments (51.89%) (*χ*^2^ = 9.078, adjusted *p* = 0.021).

^j^ Awareness of antibody development proportion of 21–30 years of employment (54.09%) was higher than ≤10 years of employment (43.88%) (*χ*^2^ = 8.382, adjusted *p* = 0.016) and 11–20 years of employment (43.34%) (*χ*^2^ = 8.410, adjusted *p* = 0.016).

^k^ Awareness of antibody development proportion of no title (34.62%) was lower than senior (51.96%) (*χ*^2^ = 13.414, adjusted *p* < 0.05) and intermediate (46.16%) (*χ*^2^ = 7.992, adjusted *p* = 0.020).

^l^ Awareness of protection duration proportion of ≥51 years old (30.70%) was lower than ≤30 years old (45.93%) (*χ*^2^ = 14.585, adjusted *p* < 0.05), 31–40 years old (46.73%) (*χ*^2^ = 18.568, adjusted *p* < 0.05) and 41–50 years old (42.64%) (*χ*^2^ = 8.770, adjusted *p* = 0.012).

^m^ Awareness of protection duration proportion of junior college or below (35.83%) was lower than bachelor’s degree (44.65%) (*χ*^2^ = 8.943, adjusted *p* = 0.009) and master’s degree or above (50.87%) (*χ*^2^ = 13.052, adjusted *p* < 0.05).

^n^ Awareness of protection duration proportion of outsourced staff (20.16%) was lower than physician (48.66%) (*χ*^2^ = 33.165, adjusted *p* < 0.05), nurse (44.31%) (*χ*^2^ = 25.856, adjusted *p* < 0.05), medical-technical staff (46.53%) (*χ*^2^ = 23.057, adjusted *p* < 0.05), administrative staff (40.00%) (*χ*^2^ = 13.957, adjusted *p* < 0.05), and the other (47.83%) (*χ*^2^ = 8.030, adjusted *p* = 0.030).

^o^ Awareness of protection duration proportion of intermediate (47.44%) was higher than no title (34.62%) (*χ*^2^ = 9.814, adjusted *p* = 0.008).

Bold values indicate statistical significance (*p* < 0.05).

### 3.2 Vaccine willingness and its influencing factors

#### 3.2.1 Willingness to vaccinate and vaccine type selection

**Figure 3:**
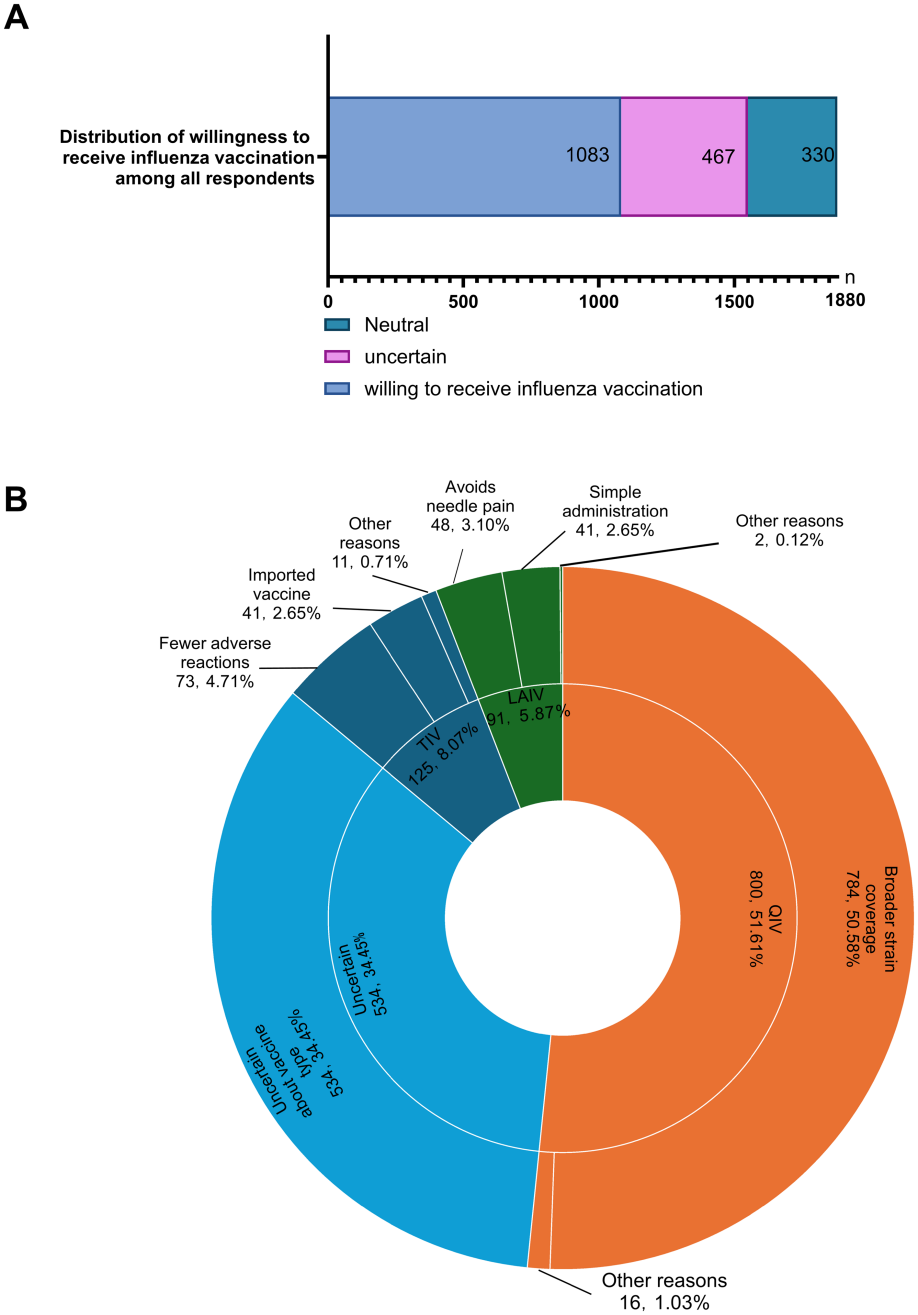
Willingness to receive influenza vaccination for the 2025 season and preferred vaccine types. **(A)** Distribution of willingness among all respondents. **(B)** Preferred influenza vaccine types among respondents who were willing or uncertain, and reasons for selection. TIV, trivalent inactivated influenza vaccine; QIV, quadrivalent inactivated influenza vaccine; LAIV, live attenuated influenza vaccine.

Before the 2025 influenza season, 1,083 respondents (57.61%) reported being willing to receive influenza vaccination, 467 (24.84%) were uncertain, and 330 (17.55%) were unwilling (Figure 3A). Among the 1,550 respondents who were willing or uncertain, 800 (51.61%) preferred the quadrivalent inactivated influenza vaccine (QIV), 125 (8.07%) preferred the trivalent inactivated influenza vaccine (TIV), 91 (5.87%) preferred the intranasal live attenuated influenza vaccine (LAIV), and 534 (34.45%) were unsure about vaccine type (Figure 3B). The main reported reason for selecting QIV was broader strain coverage; TIV was selected primarily because it was perceived to have fewer adverse reactions and was imported; and LAIV was selected to avoid needle pain and because of perceived convenience.

#### 3.2.2 Willingness to vaccinate across demographic subgroups

Willingness to receive influenza vaccination differed across demographic and workplace groups. Participants with junior college education or below had lower willingness than those with bachelor’s degree or higher. Nurses had lower willingness than physicians. Participants in tertiary MCH specialty hospitals reported lower willingness than those in secondary MCH specialty hospitals. Participants in obstetrics/gynecology reported lower willingness than those in medical-technical and outpatient/preventive-care departments. Participants without a professional title had lower willingness than those with intermediate titles (Table 7).

**Table 7:** Willingness to receive influenza vaccination for the 2025 season by characteristics (*N* = 1,880).

Characteristic	Total	Willing to vaccinate [*n* (%)]	*χ* ^2^	*p*
Sex				
Female	1,660	956 (57.59)		
Male	220	127 (57.73)	0.001	0.969
Age (years)				
≤30	455	240 (52.75)		
31–40	796	483 (60.68)		
41–50	401	238 (59.35)		
≥51	228	122 (53.51)	10.213	0.116
Education level				
Junior college or below	360	185 (51.39) ^a^		
Bachelor’s degree	1,290	755 (58.53)		
Master’s degree or above	230	143 (62.17)	12.804	**0.012**
Job category				
Physician	522	328 (62.84) ^b^		
Nurse	799	432 (54.07)		
Medical-technical staff	202	121 (59.90)		
Administrative staff	210	124 (59.05)		
Outsourced staff (e.g., cleaners)	124	66 (53.23)		
Other	23	12 (52.17)	25.722	**0.004**
Hospital level/type				
Tertiary MCH specialty hospital	1,751	987 (56.37) ^c^		
Secondary MCH specialty hospital	129	96 (74.42)	19.624	**<0.001**
Department				
Obstetrics and gynecology	298	144 (48.32) ^d^		
Pediatrics	367	218 (59.40)		
Outpatient and preventive-care departments	376	240 (63.83)		
Emergency and perioperative departments	185	98 (52.97)		
Medical-technical departments	240	148 (61.67)		
Administrative and logistics departments	272	158 (58.09)		
Other departments	142	77 (54.23)	20.902	**0.002**
Years of employment				
≤10	898	502 (55.90)		
11–20	616	373 (60.55)		
21–30	257	155 (60.31)		
≥31	109	53 (48.62)	11.361	0.078
Professional title				
Senior	281	169 (60.14)		
Intermediate	782	480 (61.38) ^e^		
Junior	635	346 (54.49)		
None	182	88 (48.35)	15.471	**0.017**

MCH, maternal and child health.

^a^ Willingness to receive influenza vaccination proportion of junior college or below (51.39%) was lower than bachelor’s degree (58.53%) (*χ*^2^ = 5.850, adjusted *p* = 0.048) and master’s degree or above (62.17%) (*χ*^2^ = 6.612, adjusted *p* = 0.030).

^b^ Willingness to receive influenza vaccination proportion of physician (62.84%) was higher than nurse (54.07%) (*χ*^2^ = 9.934, adjusted *p* = 0.012).

^c^ Willingness to receive influenza vaccination proportion of tertiary MCH specialty hospital (56.37%) was lower than secondary MCH specialty hospital (74.42%) (*χ*^2^ = 19.624, *p* < 0.001).

^d^ Willingness to receive influenza vaccination proportion of obstetrics and gynecology (48.32%) was lower than pediatrics (59.40%) (*χ*^2^ = 8.138, adjusted *p* = 0.028), outpatient and preventive-care departments (63.83%) (*χ*^2^ = 16.309, adjusted *p* < 0.05), medical-technical departments (61.67%) (*χ*^2^ = 9.539, adjusted *p* = 0.014).

^e^ Willingness to receive influenza vaccination proportion of intermediate title (61.38%) was higher than junior title (54.49%) (*χ*^2^ = 6.848, adjusted *p* = 0.036) and no title (48.35%) (*χ*^2^ = 10.355, adjusted *p* = 0.004).

Bold values indicate statistical significance (*p* < 0.05).

#### 3.2.3 Association between willingness in 2025 and vaccination behavior in 2024

Willingness to vaccinate for the 2025 season differed by vaccination behavior in 2024 (Table 8). Among participants vaccinated in 2024 (*n* = 662), 88.37% reported willingness to be vaccinated again in 2025. In contrast, among participants not vaccinated in 2024 (*n* = 1,218), only 40.89% reported willingness to vaccinate for the 2025 season.

**Table 8:** Willingness to vaccinate in 2025 by influenza vaccination status in 2024 (*N* = 1,880).

2024 vaccination status	Total	Willing [*n* (%)]	*χ* ^2^	*p*
Vaccinated	662	585 (88.37)		
Unvaccinated	1,218	498 (40.89)	404.025	<0.001

### 3.3 Knowledge, beliefs, and attitudes toward influenza vaccination

#### 3.3.1 Beliefs about influenza and influenza vaccination

**Figure 4:**
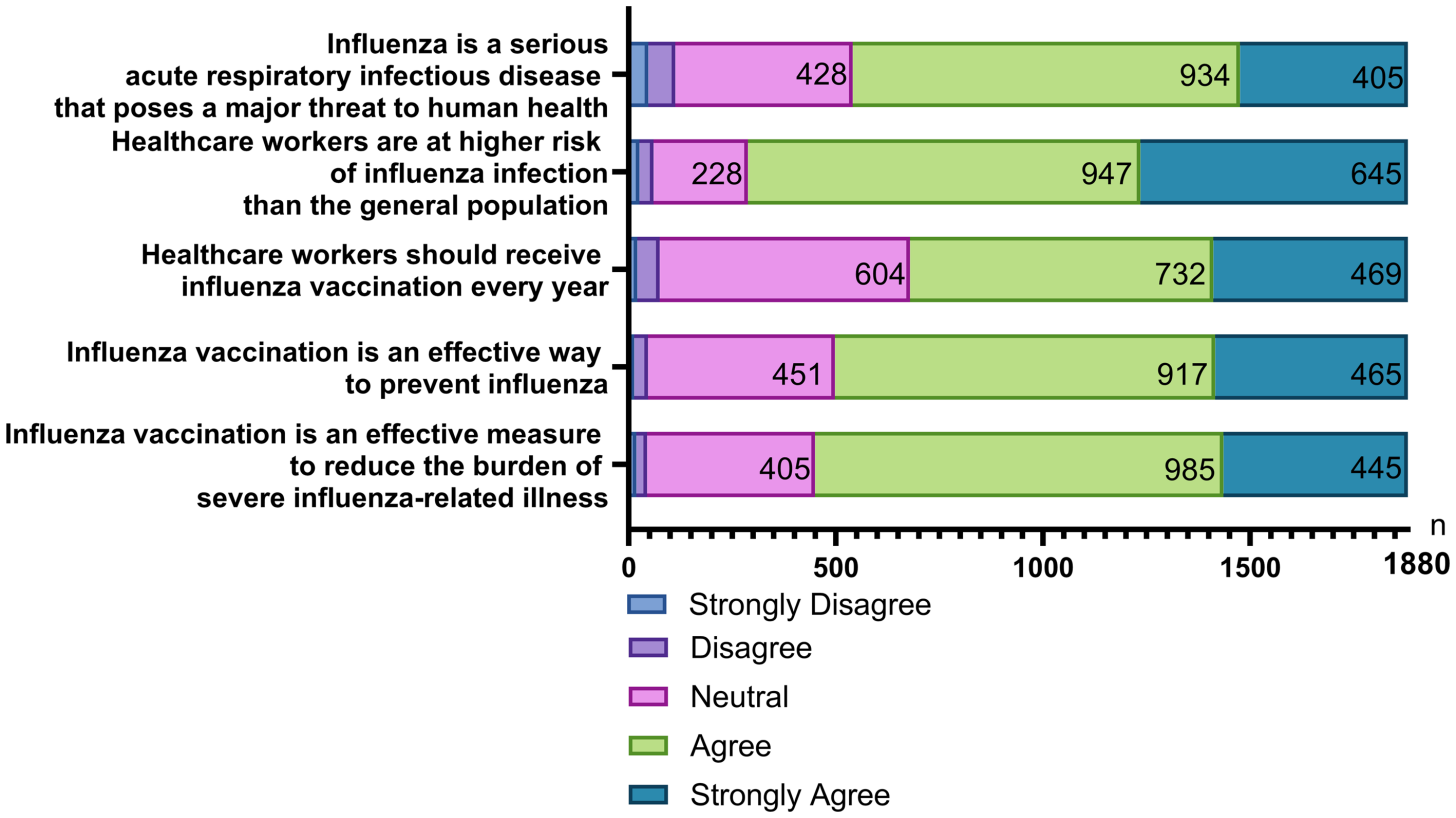
Distribution of belief scores toward influenza and influenza vaccination.

Beliefs were measured using five positively worded items spanning perceived disease risk and perceived value of vaccination (Figure 4). Observed belief scores ranged from 5 to 25 (mean, 19.74; median 20; SD 3.11) with slight negative skewness (skewness = −0.25). Belief scores differed across subgroups (Table 9). Scores were lower among participants aged 30 years or younger than those aged 31–40 and 41–50 years, and lower among participants aged 51 years or older than those aged 41–50 years. Scores increased with educational attainment and were lowest among outsourced staff. Scores were lower in tertiary MCH specialty hospitals than in secondary hospitals. Scores were lower in obstetrics/gynecology and emergency/perioperative departments than in outpatient/preventive-care departments. Participants with 10 years of employment or less had lower scores than those with 21–30 years of experience. Scores also differed by professional title, with lower scores among participants without titles.

**Table 9:** Belief scores toward influenza vaccination by characteristics (*N* = 1,880).

Characteristic	Total	Belief score	*t*/*F*	*p*
Sex				
Female	1,660	19.72 ± 3.04		
Male	220	19.83 ± 3.59	−0.425	0.671
Age (years)				
≤30	455	19.39 ± 3.14 ^a^		
31–40	796	19.91 ± 2.95		
41–50	401	20.04 ± 3.21 ^b^		
≥51	228	19.28 ± 3.33	5.744	**<0.001**
Education level				
Junior college or below	360	18.92 ± 3.23 ^c^		
Bachelor’s degree	1,290	19.81 ± 3.03 ^d^		
Master’s degree or above	230	20.63 ± 3.08	22.821	**<0.001**
Job category				
Physician	522	20.39 ± 3.08		
Nurse	799	19.34 ± 3.07 ^e^		
Medical-technical staff	202	19.94 ± 2.92		
Administrative staff	210	20.28 ± 3.14		
Outsourced staff (e.g., cleaners)	124	18.26 ± 2.94 ^f^		
Other	23	20.13 ± 3.31	14.806	**<0.001**
Hospital level/type				
Tertiary MCH specialty hospital	1,751	19.70 ± 3.12 ^g^		
Secondary MCH specialty hospital	129	20.26 ± 2.96	−1.994	**0.046**
Department				
Obstetrics and gynecology	298	19.35 ± 2.99		
Pediatrics	367	19.77 ± 3.16		
Outpatient and preventive-care departments	376	20.27 ± 3.27 ^h^		
Emergency and perioperative departments	185	19.02 ± 2.90 ^i^		
Medical-technical departments	240	20.04 ± 2.79 ^j^		
Administrative and logistics departments	272	20.01 ± 3.19 ^k^		
Other departments	142	18.92 ± 3.08	6.719	**<0.001**
Years of employment				
≤10	898	19.51 ± 3.05 ^l^		
11–20	616	19.88 ± 3.12		
21–30	257	20.11 ± 3.17		
≥31	109	19.87 ± 3.32	3.292	**0.020**
Professional title				
Senior	281	20.38 ± 3.06 ^m^		
Intermediate	782	19.86 ± 3.10 ^n^		
Junior	635	19.59 ± 3.05		
None	182	18.71 ± 3.19 ^o^	11.568	**<0.001**

MCH, maternal and child health.

^a^ Belief score toward influenza vaccination of ≤30 years old (19.39 ± 3.14) was lower than 31–40 years old (19.91 ± 2.95) (mean difference (MD) = −0.524, *p* = 0.022) and 41–50 years old (20.04 ± 3.21) (MD = −0.653, *p* = 0.016).

^b^ Belief score toward influenza vaccination of 41–50 years old (20.04 ± 3.21) was higher than ≥51 years old (19.28 ± 3.33) (MD = 0.766, *p* = 0.030).

^c^ Belief score toward influenza vaccination of junior college or below (18.92 ± 3.23) was lower than bachelor’s degree (19.81 ± 3.03) (MD = −0.890, *p* < 0.001) and master’s degree or above (20.63 ± 3.08) (MD = −1.714, *p* < 0.001).

^d^ Belief score toward influenza vaccination of bachelor’s degree (19.81 ± 3.03) was lower than master’s degree or above (20.63 ± 3.08) (MD = −0.824, *p* < 0.001).

^e^ The belief score toward influenza vaccination of nurses (19.34 ± 3.07) was lower than that of physicians (20.39 ± 3.08) (MD = −1.049, *p* < 0.001) and administrative staff (20.28 ± 3.14) (MD = −0.938, *p* = 0.002).

^f^ The belief score of outsourced staff (18.26 ± 2.94) was lower than that of physicians (20.39 ± 3.08) (MD = −2.129, *p* < 0.001), nurses (19.34 ± 3.07) (MD = −1.080, *p* = 0.003), medical-technical staff (19.94 ± 2.92) (MD = −1.678, *p* < 0.001), and administrative staff (20.28 ± 3.14) (MD = −2.018, *p* < 0.001).

^g^ The belief score of tertiary MCH specialty hospital (19.70 ± 3.12) was lower than that of secondary MCH specialty hospital (20.26 ± 2.96) (*F* = −1.994, *p* = 0.046).

^h^ The belief score toward influenza vaccination of the outpatient and preventive-care departments (20.27 ± 3.27) was higher than that of the obstetrics and gynecology departments (19.35 ± 2.99) (MD = 0.919, *p* = 0.003), emergency and perioperative departments (19.02 ± 2.90) (MD = 1.250, *p* < 0.001) and the other departments (18.92 ± 3.08) (MD = 1.349, *p* < 0.001).

^i^ The belief score of the emergency and perioperative departments (19.02 ± 2.90) was lower than the medical-technical departments (20.04 ± 2.79) (MD = −1.016, *p* = 0.006), and the administrative and logistics departments (20.01 ± 3.19) (MD = −0.993, *p* = 0.013).

^j^ The belief score of the medical-technical departments (20.04 ± 2.79) was higher than that of the other departments (18.92 ± 3.08) (MD = 1.115, *p* = 0.010).

^k^ The belief score of the administrative and logistics departments (20.01 ± 3.19) was higher than that of the other departments (18.92 ± 3.08) (MD = 1.092, *p* = 0.017).

^l^ The belief score toward influenza vaccination of the ≤10 years of employment group (19.51 ± 3.05) was lower than that of the 21–30 years group (20.11 ± 3.17) (MD = −0.598, *p* = 0.044).

^m^ The belief score toward influenza vaccination of senior title staff (20.38 ± 3.06) was higher than junior titles staff (19.59 ± 3.05) (MD = 0.784, *p* = 0.002) and no title staff (18.71 ± 3.19) (MD = 1.663, *p* < 0.001).

^n^ The belief score of intermediate title staff (19.86 ± 3.10) was higher than no title staff (18.71 ± 3.19) (MD = 1.146, *p* < 0.001).

^o^ The belief score of junior title staff (19.59 ± 3.05) was higher than no title staff (18.71 ± 3.19) (MD = 0.879, *p* = 0.006).

Bold values indicate statistical significance (*p* < 0.05).

#### 3.3.2 Attitudes toward adverse reactions after influenza vaccination

**Figure 5:**
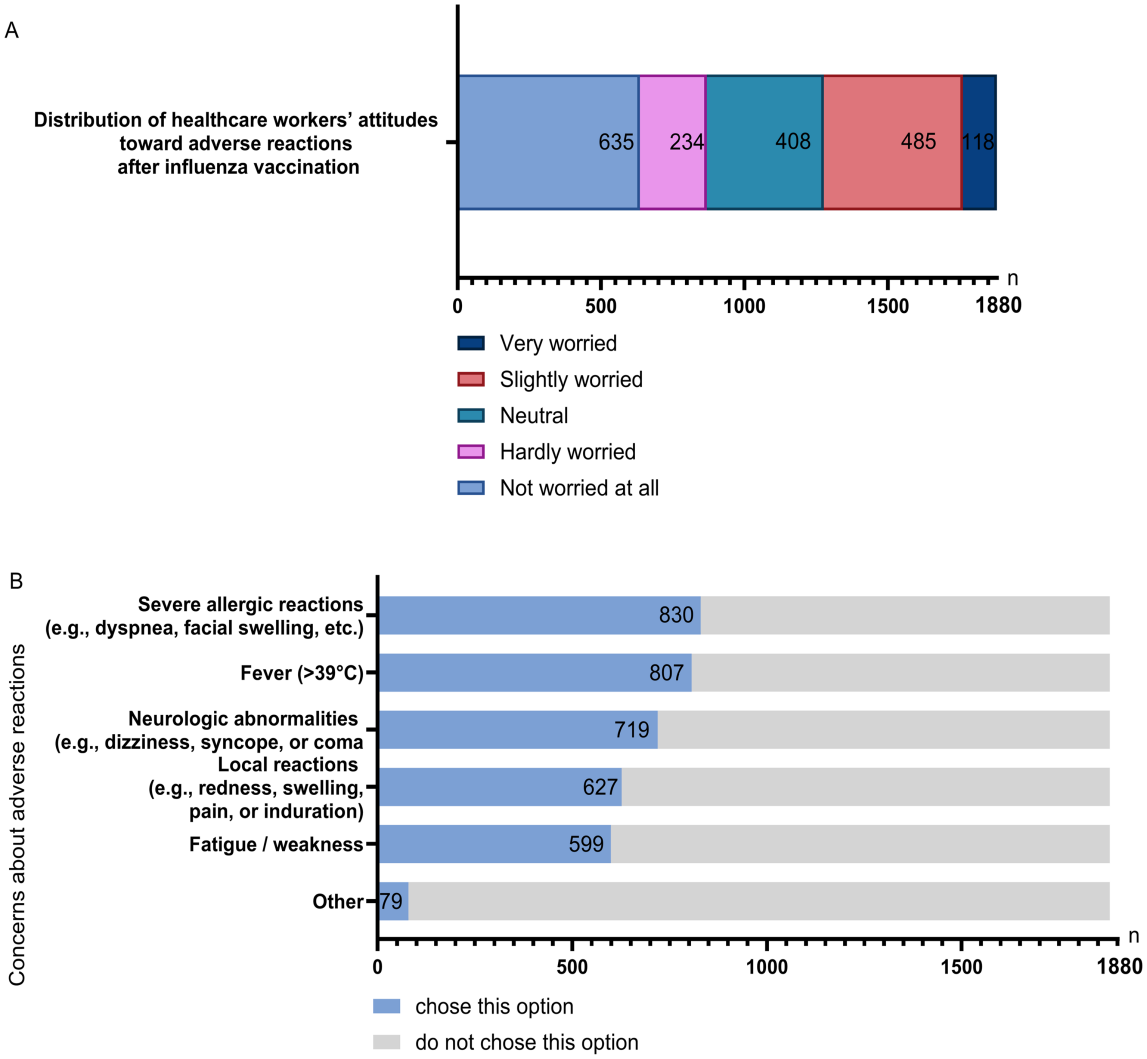
Attitudes toward adverse reactions after influenza vaccination. **(A)** Overall level of concern regarding severe adverse reactions. **(B)** Specific adverse reactions of concern reported by respondents.

Regarding concerns about serious adverse reactions after influenza vaccination, 33.77% reported not being worried at all, 12.45% were hardly worried, 21.70% were neutral, 25.80% were slightly worried, and 6.28% were very worried (Figure 5A). The most frequently reported adverse events of concern included severe allergic reactions (e.g., dyspnea, facial edema), high fever (>39 °C), neurological symptoms (e.g., dizziness, coma), local reactions (e.g., redness, swelling, pain, induration), and fatigue/weakness (Figure 5B).

#### 3.3.3 Associations between knowledge/beliefs and vaccination behavior

As Table 10, in 2024, a comparison between vaccinated and unvaccinated participants revealed that the vaccinated group had a greater awareness of antibody development time and higher belief scores than their unvaccinated counterparts (*p* < 0.05).

**Table 10:** Knowledge awareness and belief scores by influenza vaccination status in 2024 (*N* = 1,880).

Outcome	Vaccinated in 2024 (*N* = 662)	Unvaccinated in 2024 (*N* = 1,218)	*χ*^2^ or *t*	*p*
Aware of antibody development time, *n* (%)	329 (49.70)	518 (42.53)	8.905	0.003
Aware of protection duration, *n* (%)	308 (46.53)	514 (42.20)	3.261	0.071
Belief score (mean ± SD)	20.99 ± 2.92	19.05 ± 3.00	13.507	<0.001

#### 3.3.4 Factors influencing vaccination behavior

Before hierarchical logistic regression, multicollinearity among candidate predictors was examined. All variance inflation factors (VIFs) were below 5 (age: VIF = 2.155; years of employment: VIF = 2.919), indicating no concerning multicollinearity. In a linear regression model including all covariates, years of employment was not significant (*p* = 0.908), whereas age was marginally significant (*p* = 0.047). Age and years of employment were moderately correlated (Pearson *r* = 0.670, *p* < 0.001). Therefore, to reduce redundancy and improve parsimony, only age was retained in the final hierarchical logistic regression model.

Hierarchical logistic regression was used to identify factors associated with influenza vaccination uptake in 2024. The dependent variable was receipt of influenza vaccination since June 2024. Predictors were entered in three blocks: Block 1 (demographic and workplace characteristics: sex, age, education level, professional title, hospital level, department, and job category); Block 2 (knowledge variables: awareness of antibody formation time and duration of protection, plus belief score); and Block 3 (vaccination willingness). The final model was significant (chi-square = 601.416, *p* < 0.001) and explained 37.7% of the variance (Nagelkerke *R*^2^ = 0.377), with a classification accuracy of 74.8%. Adding vaccination willingness increased the model’s explanatory power by 16.8%, representing the largest incremental contribution (Table 11).

**Table 11:** Incremental contribution of blocks in the hierarchical logistic regression model (Δ*R*^2^).

Block	Variables added	*χ* ^2^	*p*	Nagelkerke *R*^2^ (cumulative)
1	Demographic characteristics	161.782	<0.001	0.113
2	Knowledge + belief score	147.219	<0.001	0.209
3	Vaccination willingness	292.415	<0.001	0.377

In the final model, older age, higher education, hospital level, department, job category, higher belief score, and vaccination willingness were independently associated with vaccination uptake (Table 12). Each additional year of age was associated with a 2.9% increase in the odds of vaccination (OR = 1.029, 95% CI: 1.009–1.049). Participants with a master’s degree or above had higher odds of vaccination than those with junior college education or below (OR = 2.067, 95% CI: 1.222–3.495). Participants working in secondary-level MCH specialty hospitals had markedly higher odds of vaccination than those in tertiary hospitals (OR = 5.377, 95% CI: 3.356–8.617). Participants in outpatient/preventive-care departments had higher odds of vaccination than those in obstetrics/gynecology (OR = 1.682, 95% CI: 1.136–2.490). Compared with physicians, nurses had higher uptake (OR = 1.804, 95% CI: 1.294–2.513), and administrative staff had substantially higher uptake (OR = 3.262, 95% CI: 1.588–6.698). Each one-point increase in belief score was associated with a 6.2% increase in the odds of vaccination (OR = 1.062, 95% CI: 1.018–1.108). Relative to participants unwilling to vaccinate, those uncertain about vaccination had higher uptake (OR = 3.431, 95% CI: 1.835–6.417), and those willing to vaccinate had markedly higher uptake (OR = 23.296, 95% CI: 12.877–42.143).

**Table 12:** Hierarchical multivariable logistic regression for influenza vaccination uptake in 2024.

Variable	*β*	Wald	*p*	OR (95% CI)
Sex (male)	0.268	1.948	0.163	1.307 (0.897 ~ 1.904)
Age (years)	0.028	7.857	0.005 ^a^	1.029 (1.009 ~ 1.049)
Education level		7.669	0.022 ^b^	
Junior college or below				
Bachelor’s degree	0.285	2.186	0.139	1.329 (0.911 ~ 1.939)
Master’s degree or above	0.726	7.340	0.007	2.067 (1.222 ~ 3.495)
Professional title		3.147	0.370	
None				
Junior	0.077	0.081	0.775	1.080 (0.635 ~ 1.837)
Intermediate	0.312	1.191	0.275	1.366 (0.780 ~ 2.390)
Senior	0.432	1.586	0.208	1.541 (0.786 ~ 3.018)
Hospital level (secondary)	1.682	48.896	<0.001 ^c^	5.377 (3.356 ~ 8.617)
Department		16.534	0.011 ^d^	
Obstetrics and gynecology				
Pediatrics	0.173	0.733	0.392	1.188 (0.801 ~ 1.764)
Outpatient and preventive-care departments	0.520	6.755	0.009	1.682 (1.136 ~ 2.490)
Emergency and perioperative departments	−0.026	0.012	0.914	0.974 (0.605 ~ 1.569)
Medical-technical departments	0.131	0.251	0.617	1.140 (0.683 ~ 1.902)
Administrative and logistics departments	−0.043	0.016	0.899	0.957 (0.491 ~ 1.868)
Other departments	−0.433	2.269	0.132	0.648 (0.369 ~ 1.139)
Job category		17.458	0.004 ^e^	
Physician				
Nurse	0.590	12.143	<0.001	1.804 (1.294 ~ 2.513)
Medical-technical staff	0.254	1.093	0.296	1.290 (0.801 ~ 2.077)
Outsourced staff (e.g., cleaners)	0.523	1.782	0.182	1.687 (0.783 ~ 3.636)
Administrative staff	1.182	10.370	0.001	3.262 (1.588 ~ 6.698)
Other job types	0.522	0.855	0.355	1.685 (0.558 ~ 5.096)
Aware of antibody development time	0.079	0.438	0.508	1.082 (0.857 ~ 1.365)
Aware of protection duration	0.061	0.268	0.605	1.063 (0.844 ~ 1.339)
Belief score	0.060	7.646	0.006 ^f^	1.062 (1.018 ~ 1.108)
Vaccination willingness		211.395	<0.001 ^g^	
Unwilling				
Uncertain	1.233	14.901	<0.001	3.431 (1.835 ~ 6.417)
Willing	3.148	108.343	<0.001	23.296 (12.877 ~ 42.143)

^a^ Each additional year of age was associated with higher odds of influenza vaccination uptake.

^b^ Compared with junior college or below, participants with a master’s degree or above had higher odds of influenza vaccination uptake.

^c^ Participants working in secondary MCH specialty hospitals had higher odds of influenza vaccination uptake than those in tertiary MCH specialty hospitals.

^d^ Compared with obstetrics and gynecology, participants in outpatient and preventive-care departments had higher odds of influenza vaccination uptake.

^e^ Compared with physicians, nurses and administrative staff had higher odds of influenza vaccination uptake.

^f^ Each one-point increase in belief score was associated with higher odds of influenza vaccination uptake.

^g^ Compared with participants unwilling to vaccinate, those who were uncertain or willing had higher odds of influenza vaccination uptake.

### 3.4 Analysis and summary of special groups

#### 3.4.1 Vaccination among pregnant women

**Figure 6:**
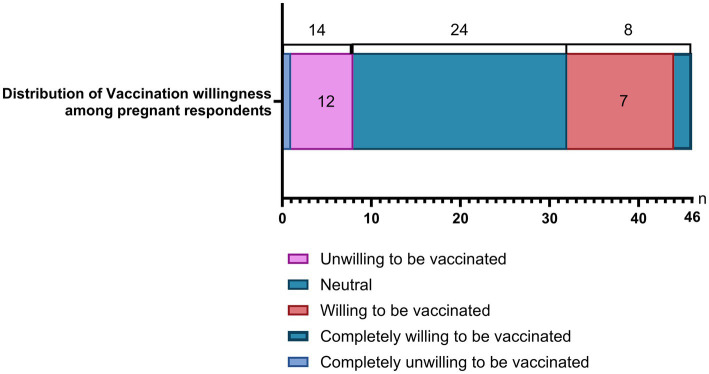
Willingness to receive influenza vaccination among pregnant respondents.

Among 46 pregnant respondents, 12 reported being willing and 2 reported being fully willing to receive influenza vaccination, yielding 14 respondents (30.44%) who were willing or fully willing to vaccinate. Twenty-four respondents (52.17%) reported neutral attitudes. Eight respondents (17.39%) reported being unwilling or completely unwilling to receive vaccination (Figure 6).

## 4 Discussion

This cross-sectional study surveyed 1,880 healthcare workers from 10 MCH institutions in Chengdu. Participants were predominantly female and primarily from tertiary institutions, representing a range of departments including outpatient/preventive-care, pediatrics, obstetrics/gynecology, medical-technical, and administrative/logistics units. The study provides a comprehensive characterization of post-pandemic influenza vaccination uptake, knowledge and beliefs, and willingness to vaccinate in MCH settings, and identifies factors associated with vaccination behavior.

Influenza vaccination uptake among healthcare workers in Chengdu MCH institutions was 35.21%, consistent with prior studies in comparable settings (11, 12). Willingness to receive influenza vaccination before the 2025 season was 57.61%, indicating a notable intention-behavior gap (13, 14). This suggests that, beyond individual attitudes, organizational and contextual barriers remain salient in MCH environments. Outpatient surges, shift work, and perioperative workload may increase opportunity costs and impede conversion of intention into behavior (15).

Vaccination history was strongly related to future willingness: 88.37% of those vaccinated in 2024 reported willingness to vaccinate again in 2025 (16). In contrast, only 40.89% of those unvaccinated in 2024 reported willingness for the next season, suggesting persistent barriers among first-time non-vaccinators. The most common reasons for non-vaccination included perceiving oneself as healthy, time constraints, concerns about adverse reactions, doubts about vaccine effectiveness due to viral mutation, and perceived lack of benefit from prior vaccination.

We observed positive associations between age and education and vaccination behavior, consistent with previous evidence. Older healthcare workers may perceive greater personal risk from influenza and its complications, and individuals with higher educational attainment may have stronger confidence in vaccine effectiveness and safety. These findings underscore the role of knowledge and risk appraisal in health decision-making (17, 18). Uptake also differed by job category (19). In this study, nurses and administrative staff had higher vaccination uptake than physicians, with particularly high uptake among administrative staff. This pattern may reflect differential responsiveness to institutional policies, scheduling constraints, and workflow structure across roles (20).

Despite generally favorable attitudes, substantial gaps in objective knowledge were identified. Only 45.05% correctly identified the 2–4 week antibody development window, and knowledge of protection duration was similarly limited. Stratified analyses showed gradients by education and professional title, with the lowest awareness among outsourced staff. These structural gaps may reflect unequal access to training and guideline updates across employment categories.

Belief scores differed across institutional and departmental contexts. Scores were lower in obstetrics/gynecology and emergency/perioperative departments than in outpatient/preventive-care and medical-technical departments, and lower in tertiary than secondary hospitals. High workload and complex clinical processes may increase perceived costs and reduce the perceived value of vaccination (21). Notably, reported post-vaccination discomfort was mostly mild and transient, yet concerns about severe adverse events persisted for some respondents. Transparent communication about typical reactions, along with on-site adverse-event monitoring and feedback, may help align risk perceptions with observed experiences and improve trust (22).

Hierarchical logistic regression demonstrated acceptable model performance. The most salient finding was the strong contribution of vaccination willingness, which substantially increased model explanatory power. This supports willingness as a proximal determinant of vaccination behavior. Belief score was independently associated with uptake, reinforcing a pathway in which knowledge informs beliefs, beliefs shape willingness, and willingness drives behavior (23, 24). The objective knowledge variables were not significant after beliefs and willingness were included, suggesting that knowledge may influence behavior largely through beliefs and willingness rather than directly.

Institutional and departmental differences also highlight the importance of organizational capacity and convenience. Secondary hospitals may achieve higher coverage more readily due to simpler processes or more centralized campaigns, whereas tertiary institutions may face greater workflow fragmentation. Interventions should therefore combine content-focused strategies (improving key knowledge points and confidence) with system-focused strategies (flexible scheduling, on-site vaccination clinics, reminders, and streamlined procedures).

Vaccination in pregnancy remains a critical gap. Only around one-third of pregnant respondents reported being willing to be vaccinated, which diverges from guideline recommendations that prioritize pregnancy as a key indication (25, 26). Potential drivers include persistent safety concerns, insufficient pregnancy-specific counseling, and limited service accessibility. Targeted training for healthcare workers and tailored communication to pregnant women are needed to improve confidence and uptake.

A number of limitations merit consideration. This survey was conducted at 10 MCH institutions in Chengdu and the sample was dominated by tertiary hospitals; as a result, the findings primarily reflect the local MCH context and may not directly generalize to other regions or primary-level settings. The questionnaire was disseminated through institutional WeChat working groups and the response rate was 46.48%, so systematic differences between respondents and non-respondents (e.g., workload, interest in vaccination) may have influenced the estimates. Although multiple institutions were included, staff within the same hospital or department often share similar policies, training exposure, and access to vaccination services, implying within-unit clustering; without additional adjustment (e.g., multilevel modeling or cluster-robust inference), standard errors may be underestimated and some associations may be overstated. Moreover, several organizational determinants could not be captured or quantified, such as whether vaccination was free/subsidized, the availability and operating hours of on-site clinics, scheduling support, reminder/mobilization practices, and links to managerial requirements. Residual confounding may therefore remain, and differences by hospital level or department should be interpreted alongside institutional processes and access. Subgroup analyses were limited by small numbers in certain groups (e.g., outsourced staff, males, and pregnant respondents), and these results are best viewed as descriptive. Finally, the cross-sectional design and self-reported infection/vaccination history may involve recall error and do not support causal inference. Future studies could broaden institutional and geographic coverage, incorporate organizational-level measures and objective vaccination records where feasible, and apply multilevel or longitudinal designs to strengthen inference and clarify mechanisms.

## 5 Conclusion

Influenza vaccination coverage among healthcare workers in Chengdu MCH institutions was moderate and remained suboptimal in the post-pandemic period, with a substantial intention-behavior gap. Uptake was primarily associated with beliefs and willingness, as well as occupational role and institutional context. Knowledge deficits, concerns about adverse reactions, and logistical barriers likely contribute to low coverage. To protect pregnant women and infants and reduce nosocomial transmission, interventions should strengthen confidence and pregnancy-specific counseling, improve convenience through flexible scheduling and on-site vaccination, and tailor strategies to departments and job categories with lower uptake.

## Data Availability

The raw data supporting the conclusions of this article will be made available by the authors, without undue reservation.

## Supplementary material

SUPPLEMENTARY TABLE 1 Questionnaire used in this study.
