# Supplementary material for: Influenza vaccination uptake and associated factors among healthcare workers in Chengdu’s maternal and child health institutions in the post-pandemic era
Source: Front Public Health. 2026 Apr 13;14:1766101. doi: 10.3389/fpubh.2026.1766101 (PMC13111301; doi:10.3389/fpubh.2026.1766101)
Supplement: SUPPLEMENTARY TABLE 1 — Questionnaire used in this study. [file Table_1.docx]

**Survey on Influenza Vaccination Knowledge, Status, and Intentions among Hospital Staff**

This questionnaire aims to understand hospital staff's knowledge, current vaccination status, and future intentions regarding the influenza vaccine, to better plan vaccination initiatives. Participation in this survey is entirely voluntary. Your responses will be kept strictly confidential. You may withdraw or skip questions at any time. Completing the questionnaire implies your informed consent to participate. Thank you for your support!

1. Name of your hospital: (Open-ended)

_________________________________

1. Your department: (Single choice)

○ Pediatrics (Internal Medicine)

○ Pediatrics (Surgery)

○ Emergency Department

○ Traditional Chinese Medicine Department

○ Intensive Care Unit (ICU)

○ Obstetrics and Gynecology

○ Medical Technology Departments (e.g., Lab, Radiology)

○ Outpatient and Preventive Healthcare Departments

○ Administrative and Logistics Departments

○ Other, please specify _________________

1. Gender: (Single choice)

○ Male

○ Female

1. Age (years): (Open-ended)

_________________________________

1. Position/Job Type: (Single choice)

○ Physician

○ Nurse

○ Resident/Intern/Trainee Physician

○ Resident/Intern/Trainee Nurse

○ Medical Technician

○ Outsourced Staff (e.g., cleaning)

○ Administrative Staff

○ Medical Assistant

1. Years of Work Experience (years): (Open-ended)

_________________________________

1. Education Level: (Single choice)

○ Junior College or below

○ Bachelor's Degree

○ Master's Degree or above

1. Professional Title: (Single choice)

○ Senior

○ Intermediate

○ Junior

○ None

1. Are you concerned about experiencing severe adverse reactions after receiving the influenza vaccine? (Single choice)

○ Not concerned at all

○ Slightly unconcerned

○ Neutral

○ Slightly concerned

○ Very concerned

1. Which of the following adverse reactions are you concerned about after influenza vaccination? (Multiple choice)

□ Severe allergic reaction (e.g., difficulty breathing, facial swelling)

□ High fever (over 39°C)

□ Neurological abnormalities (e.g., dizziness, loss of consciousness)

□ Local reaction (e.g., redness, swelling, pain, or induration)

□ Fatigue, weakness

□ Other _________________

□ I am not concerned about any side effects

1. Have you received the influenza vaccine since June 2024? (Single choice)

○ Yes

○ No

1. If you received the influenza vaccine in 2024, did you experience any discomfort? (Single choice)

○ Yes _________________

○ No

1. Reasons for NOT receiving the influenza vaccine in 2024: (Multiple choice)

□ Believe personal physical health is good, not needed

□ Have doubts about vaccine effectiveness

□ Previously vaccinated but effect was not noticeable

□ Influenza virus mutates quickly, vaccine effectiveness is poor

□ Concerned about vaccine adverse reactions

□ No time to get vaccinated

□ Influenza is not severe in my province

□ Don't know where to get vaccinated

□ Have contraindications

□ Pregnant/Planning pregnancy/Breastfeeding

□ Allergic to the vaccine

□ Already had influenza

□ Acute infection period

□ Other _________________

1. Since June 2024, have you been infected with influenza? (Single choice)

○ Yes

○ No

○ Unsure, had influenza-like symptoms but no test done

1. When did symptoms or infection occur? (Single choice)

○ After influenza vaccination

○ Before influenza vaccination

1. Did you get tested for the influenza virus? (Single choice)

○ Yes

○ No

1. Influenza test result: (Single choice)

○ Negative

○ Positive

1. Were you hospitalized due to influenza-like illness or influenza? (Single choice)

○ Yes

○ No

1. Influenza is a serious acute respiratory infectious disease that poses a significant hazard to human health. (Single choice)

○ Strongly disagree

○ Disagree

○ Neutral

○ Agree

○ Strongly agree

1. Healthcare workers are at greater risk of influenza infection (compared to the general population). (Single choice)

○ Strongly disagree

○ Disagree

○ Neutral

○ Agree

○ Strongly agree

1. Healthcare workers should receive the influenza vaccine annually. (Single choice)

○ Strongly disagree

○ Disagree

○ Neutral

○ Agree

○ Strongly agree

1. Influenza vaccination is an effective measure to prevent influenza. (Single choice)

○ Strongly disagree

○ Disagree

○ Neutral

○ Agree

○ Strongly agree

1. After influenza vaccination, how long does it typically take to develop protective levels of antibodies? (Single choice)

○ Within 1 week

○ 2-4 weeks

○ 1 month

○ 2 months

○ Unsure

1. Influenza vaccination is an effective means to reduce the burden of severe influenza-related outcomes. (Single choice)

○ Strongly disagree

○ Disagree

○ Neutral

○ Agree

○ Strongly agree

1. Are you willing to receive the influenza vaccine before the 2025 flu season? (Single choice)

○ Yes

○ No

○ Unsure

1. Are you aware of the different types of influenza vaccines? (Single choice)

○ Yes, I know the difference between trivalent and quadrivalent vaccines

○ No, I am not clear about the types of influenza vaccines

○ Partially aware

1. Are you aware of the updating process for influenza vaccines? (Single choice)

○ Yes, I know the influenza vaccine is updated annually based on virus variations

○ No, I am not aware of the updating process

○ Partially aware

1. Are you aware that the protection period after influenza vaccination is approximately ( ) months? (Open-ended)

_________________________________

1. For the 2025 influenza vaccine, which type would you choose? (Single choice)

○ Trivalent influenza vaccine

○ Quadrivalent influenza vaccine

○ Nasal spray influenza vaccine (live attenuated)

○ Unsure

1. Your reason for choosing the quadrivalent vaccine: (Single choice)

○ Protects against more virus types

○ Other _________________

1. Your reason for choosing the trivalent vaccine: (Single choice)

○ Imported vaccine

○ Fewer adverse reactions

○ Other _________________

1. Your reason for choosing the nasal spray influenza vaccine (live attenuated): (Single choice)

○ Avoids needle pain, no injection needed

○ Simple administration procedure, no specialized injection training required

○ Other (please specify) _________________

1. Are you aware of the "Chinese Influenza Vaccination Technical Guidelines for 2024" issued by the Chinese Center for Disease Control and Prevention? (Single choice)

○ Completely unaware

○ Slightly aware

○ Unsure

○ Somewhat aware

○ Fully aware

1. Through which channels do you obtain information about influenza vaccines? (Multiple choice)

□ Internet

□ Doctor's recommendation

□ Colleagues, family, or friends

□ Medical books

□ Internal hospital training

□ Hospital promotional materials

□ Other _________________

1. Are you pregnant? (Single choice)

○ Yes

○ No

1. Do you think pregnant women can receive the influenza vaccine? (Single choice)

○ Yes

○ No

1. Are you willing to receive the influenza vaccine if recommended by a doctor? (Single choice)

○ Completely unwilling

○ Unwilling

○ Neutral

○ Willing

○ Completely willing
